# Transcriptomic analysis of the adaptation to prolonged starvation of the insect-dwelling *Trypanosoma cruzi* epimastigotes

**DOI:** 10.3389/fcimb.2023.1138456

**Published:** 2023-04-06

**Authors:** Pablo Smircich, Leticia Pérez-Díaz, Fabricio Hernández, María Ana Duhagon, Beatriz Garat

**Affiliations:** ^1^Sección Genómica Funcional, Facultad de Ciencias, Universidad de la República, Montevideo, Uruguay; ^2^Laboratorio de Bioinformática, Departamento de Genómica, Instituto de Investigaciones Biológicas Clemente Estable, Montevideo, Uruguay; ^3^Departamento de Genética, Facultad de Medicina Universidad de la República, Montevideo, Uruguay

**Keywords:** *Trypanosoma cruzi*, life cycle, transcriptomics, starvation, differentiation

## Abstract

*Trypanosoma cruzi* is a digenetic unicellular parasite that alternates between a blood-sucking insect and a mammalian, host causing Chagas disease or American trypanosomiasis. In the insect gut, the parasite differentiates from the non-replicative trypomastigote forms that arrive upon blood ingestion to the non-infective replicative epimastigote forms. Epimastigotes develop into infective non-replicative metacyclic trypomastigotes in the rectum and are delivered *via* the feces. In addition to these parasite stages, transitional forms have been reported. The insect-feeding behavior, characterized by few meals of large blood amounts followed by long periods of starvation, impacts the parasite population density and differentiation, increasing the transitional forms while diminishing both epimastigotes and metacyclic trypomastigotes. To understand the molecular changes caused by nutritional restrictions in the insect host, mid-exponentially growing axenic epimastigotes were cultured for more than 30 days without nutrient supplementation (prolonged starvation). We found that the parasite population in the stationary phase maintains a long period characterized by a total RNA content three times smaller than that of exponentially growing epimastigotes and a distinctive transcriptomic profile. Among the transcriptomic changes induced by nutrient restriction, we found differentially expressed genes related to managing protein quality or content, the reported switch from glucose to amino acid consumption, redox challenge, and surface proteins. The contractile vacuole and reservosomes appeared as cellular components enriched when ontology term overrepresentation analysis was carried out, highlighting the roles of these organelles in starving conditions possibly related to their functions in regulating cell volume and osmoregulation as well as metabolic homeostasis. Consistent with the quiescent status derived from nutrient restriction, genes related to DNA metabolism are regulated during the stationary phase. In addition, we observed differentially expressed genes related to the unique parasite mitochondria. Finally, our study identifies gene expression changes that characterize transitional parasite forms enriched by nutrient restriction. The analysis of the here-disclosed regulated genes and metabolic pathways aims to contribute to the understanding of the molecular changes that this unicellular parasite undergoes in the insect vector.

## Introduction

*Trypanosoma cruzi* is a digenetic parasitic kinetoplastid that alternates between an invertebrate host—Triatominae vector—and a mammalian host and causes Chagas disease or American trypanosomiasis (Chagas, 1909), a life-threatening problem currently affecting 6-7 million people mainly in endemic areas of Latin America (https://www.who.int/news-room/fact-sheets/detail/chagas-disease-(american-trypanosomiasis). *T. cruzi* has been classified in several Discrete Typing Units (DTUs, TcI to TcVI and TcBat). This genetic diversity is associated with differences in virulence heterogeneity, geographical distribution, insect host and is differentially sampled in domestic and/or sylvatic cycles (Noireau et al., 2009; Brenière et al., 2016). Since the parasite can infect a wide range of mammalian hosts that can be considered urban reservoirs, zoonotic transmission has been recognized as a public health issue (Urdaneta-Morales, 2014). In humans, though the infection can be achieved through blood transfusions or organ transplant, congenitally from mother to child, or by accidental ingestion of contaminated food, *T. cruzi* is mainly transmitted to mammals by blood-sucking insects of the subfamily Triatominae widely distributed in Latin America and currently expanding from rural to urban areas (Alarcón de Noya et al., 2022).

In the vector, the non-replicative *T. cruzi* trypomastigotes that arrive with blood ingestion differentiate from the non-infective epimastigote forms, which actively replicate in the vector’s midgut and progress through the rectum developing the infective non-replicative metacyclic trypomastigotes. In addition, other parasite forms have been recognized in the digestive tract of the insect and in the *in vitro* metacyclogenesis process, which was later confirmed (Kollien and Schaub, 2000; Tyler and Engman, 2001; Nepomuceno-Mejía et al., 2010; Shaw et al., 2016; Gonçalves et al., 2018; De Souza and Barrias, 2020), and recently designated as transitional epimastigotes (De Souza and Barrias, 2020).

A role for these forms as a stage in the life cycle of *T. cruzi* developed in response to nutrient availability in the insect intestinal environment was early proposed (Kollien and Schaub, 1998). Indeed, the long starvation periods that follow the abundant blood ingestion of triatomine sharply affect not only parasite population density but also the proportion of developmental stages, increasing the proportion of transitional forms while decreasing metacyclic trypomastigotes and epimastigotes in insects starved for more extended periods (Kollien and Schaub, 1998). It is considered that the triatomine nutritional behavior derives from the fact that having few meals but of large blood amounts minimizes the danger that the vertebrate may cause to blood-sucking insects (Sterkel et al., 2017). Considering not only morphological changes but also transcriptional and post-transcriptional events, mature stationary phase epimastigotes have been proposed as a distinctive pre-adaptive stage with the ability to differentiate into the metacyclic form or to return to the replicative epimastigote stage depending on the availability of nutrients (Hernández et al., 2012). More recently, the transitional epimastigote has also been claimed as a distinctive developmental stage (De Souza and Barrias, 2020). Understanding developmental processes in the insect host has proven to be a challenging task. Several factors that influence this process *in vivo* cannot be readily reproduced on *in-vitro* models. Emerging technologies of tissue and 3D cell culture may overcome some of these issues. Indeed, pharmacological analysis for the prevention and treatment of diseases caused by unicellular parasites is being improved using these methods to model host-parasite interactions (Pance, 2021). In *T. cruzi* ony a few reports using these methodologies have been described in the literature and are limited to mammalian stages mostly in cardiac tissues (da Silva Lara et al., 2018; Bozzi et al., 2019; Sass et al., 2019a; Sass et al., 2019b). The relevance of studying host-parasite interactions also in mammalian gastrointestinal tissue has been recognized (Breyner et al., 2020). It would be interesting to develop such approaches by using insect-derived cells. While these emerging strategies will necessarily improve the current understanding of parasite-insect-host interactions, reductionist *in vitro* approaches are still valuable tools to gain insight into the molecular mechanisms involved.

Despite its pervasive constitutive transcription, *T. cruzi* exhibits exceptional metabolic flexibility to respond to environmental stress. Proteomic and transcriptome studies have shown that the parasites display gene expression changes when entering the stationary growth phase. Differential expression of genes related to the cell cycle, pathogenesis, and metabolic processes (Santos et al., 2018) or proteins related to replication status and autophagy (Avila et al., 2018) have been found. Using metabolomics targeted at energy metabolism and oxidative imbalance, a finely tuned metabolic switch from glucose to amino acid consumption has been revealed at the beginning of the stationary growth phase (Barisón et al., 2017). However, the effects of prolonged nutrient restriction on insect-dwelling parasite forms have only been partially accounted for. Using an approach of sudden nutrient restriction to the epimastigotes reaching the stationary growth phase, the study of the impact on the unique mitochondria of *T. cruzi* epimastigotes revealed structural changes, organelle swelling and impaired oxidative phosphorylation with increased ROS levels and overexpressed antioxidant enzymes, as well as an exacerbated expression of different autophagy-related genes (Pedra-Rezende et al., 2021). In addition, the alkalinization of acidocalcisomes through histidine ammonia-lyase has recently been demonstrated to be essential for survival under starvation conditions (Mantilla et al., 2021). Besides, prolonged nutrient starvation of *T. cruzi* epimastigotes increased mitochondrial rRNAs and some mRNAs (Shaw et al., 2016)in different strains (Gerasimov et al., 2022). Interestingly, while mitochondrial-encoded respiratory complex subunit mRNA abundances also increase, a similar pattern was not found for the nuclear-encoded subunit mRNAs (Shaw et al., 2016).

To understand the molecular changes derived from the nutritional restrictions that *T. cruzi* slowly undergoes in the insect vector, we here present and analyze the transcriptomes obtained from axenic epimastigotes cultured *in vitro* for a prolonged period without further nutrient supplementation. Although the parasite population in the prolonged stationary phase cannot be considered homogenous, we found that it remains molecularly uniform and differs from exponentially growing epimastigotes. This parasite population is characterized by a total RNA per cell content of approximately one-third of that of exponentially growing parasites and a distinctive transcriptomic profile. Besides the expected expression changes of genes related to surface proteins (Santos et al., 2018), the persistent differentially expressed genes (DEGs) could be categorized in three GO terms: vacuoles, including reservosome and contractile vacuole, chromosome, and the kinetoplast. The analysis of the DEGs suggests a role for intracellular organelles in the adaptation to starving conditions and derived changes in the culture through a quiescent status preserving metabolic homeostasis and osmoregulation. Finally, we identified genes whose expression profiles cannot be attributed to the differentiation to infective metacyclic trypomastigotes (metacyclogenesis), thus could be delineating the molecular markers of the transitional parasite forms induced by nutrient restriction.

## Materials and methods

### Parasite culture

Epimastigotes of the *T. cruzi* Dm28c strain (TcI DTU) (Contreras et al., 1988) were cultured at 28°C in fresh BHI medium (Oxoid) supplemented with heat-inactivated 10% Fetal Bovine Serum (Capricorn) and penicillin (100 units/mL) and streptomycin (100 μg/mL). The parasite culture was initiated by 1x10^6^ cells/mL previously maintained in the mid-exponential growth phase through continuous dilutions. Three biological replicates were assessed. For each independent experiment, parasites were counted directly by light microscopy using a Neubauer chamber in triplicate. Metacyclic trypomastigotes were distinguished based on morphological features and relative nucleus to kinetoplast location using DAPI- and HE-stained PFA-fixed parasites. Several images were acquired, and at least 100 cells were counted for each independent triplicate experiment.

### RNA extraction and evaluation

Total RNA was extracted from 2x10^7^ parasites using Trizol reagent (Life Technologies) as described by the manufacturer. RNA cleanup was performed using DNA-free kit (Life Technologies) according to the manufacturer’s instructions. Purified RNA was quantified using Nanodrop by evaluating the absorbance at 260 and 280 nm, and the quality and integrity were assessed using an Agilent RNA 6000 Nano Chip on a Bioanalyzer 2100 (Agilent Technologies).

### Transcriptomic assays and data analysis

Three independent replicates of each condition were sequenced at Macrogen using Illumina TruSeq™ RNA Sample Preparation Kit v2 and HiSeq 2500 (http://www.macrogen.com). Trimmomatic (Bolger et al., 2014) was used to obtain high-quality reads that were mapped to the Esmeraldo-like *T. cruzi* genome (version 29, http://tritrypdb.org) using bowtie2 in –very sensitive mode (Langmead and Salzberg, 2012). The number of reads per gene was determined using htseq-count (Anders et al., 2015). For further analysis, only genes with at least 0.5 counts per million in at least one sample were kept. Between 5.8 and 8.9 million reads were mapped per sample. Nine thousand seven hundred seventy-six genes met our exclusion criteria and were used for further analysis. For all pairwise comparisons, differentially expressed genes were assessed using the DEseq2 package available in R (Love et al., 2014). To determine differentially expressed genes (DEGs), a log2 fold change (FC) of |1| and a false discovery rate (FDR) less than 0.05 was considered. Overrepresentation of GO terms among DEGs lists was established using Cluster Profiler in R (Wu et al., 2021), setting a Bonferroni adjusted p-value of less than 0.1 as a cutoff for significance. Statistical analyses and plots were performed in R unless otherwise specified.

## Results and discussion

### Prolonged *in vitro* culture of *T. cruzi* epimastigotes without nutrient renewal leads to a decay of total RNA per cell content

Parasite life cycle depends on complex interactions with biological, chemical and physical factors in the natural host. The host microbiome, temperature and nutritional environment including the contribution of parasite autophagy have been recognized as relevant variables that affect differentiation (Jimenez et al., 2008; Li et al., 2011; Díaz-Albiter et al., 2016; Teotônio et al., 2019; Cruz-Saavedra et al., 2020; Paes et al., 2020).

In order to study the transcriptomic changes provoked by the nutrient restriction that *T. cruzi* encounters in the bug´s digestive tract, we initiated *in vitro* cultures with mid-log phase epimastigotes in complete cell culture media and allowed them to grow without medium replenishment. Under these conditions, parasites actively replicate and grow exponentially at day 7 (E), reaching stationary growth on day 14 (early stationary phase, Se). Although the cultures were maintained until day 35, a decline of the growth plateau was evident after day 30. Considering the observed growth curve, in addition to the parasite populations mentioned above (E and Se), we analyzed parasites on day 21 (intermediate stationary phase, Si) and day 28 (final stationary phase, Sf) to evaluate the effects of prolonged culture without medium replenishment.

Parasite morphology was used as a phenotypic marker of the developmental stage. Either metacyclic trypomastigotes, whose presence at low proportion in epimastigote cultures is widely recognized, and transitional forms also previously reported were observed. The percentage of metacyclic trypomastigotes that was detected since the very beginning at low percentages (below 10%), at least untill the intermediate stationary phase plateau (3.8 ± 1.7%, 5.4 ± 0.4% and 7.9 ± 0.6% for E, Se and Si respectively), sharply surpassed 30% at the end of the stationary phase (32.1 ± 5.4% for Sf). Given the characteristics of the transitional forms (displaying a positioning of the kinetoplast, flagellum, and nucleus corresponding to the epimastigote stage but with non-classical epimastigote morphology of the cell body and flagellum) they cannot be precisely quantified by visual inspection (Tyler and Engman, 2001; Nepomuceno-Mejía et al., 2010; Shaw et al., 2016; Gonçalves et al., 2018; De Souza and Barrias, 2020). Nevertheless, enrichment of transitional forms was observed in the stationary phase (Se, Si, and Sf). It has been proposed that flagellar elongation may provide an extended surface for nutrient uptake in unfavorable nutrient conditions (Tyler and Engman, 2001). Meanwhile, repositioning of the kinetoplast has only been observed in the later stages of the metacyclogenesis process (Gonçalves et al., 2018).

**Figure 1:**
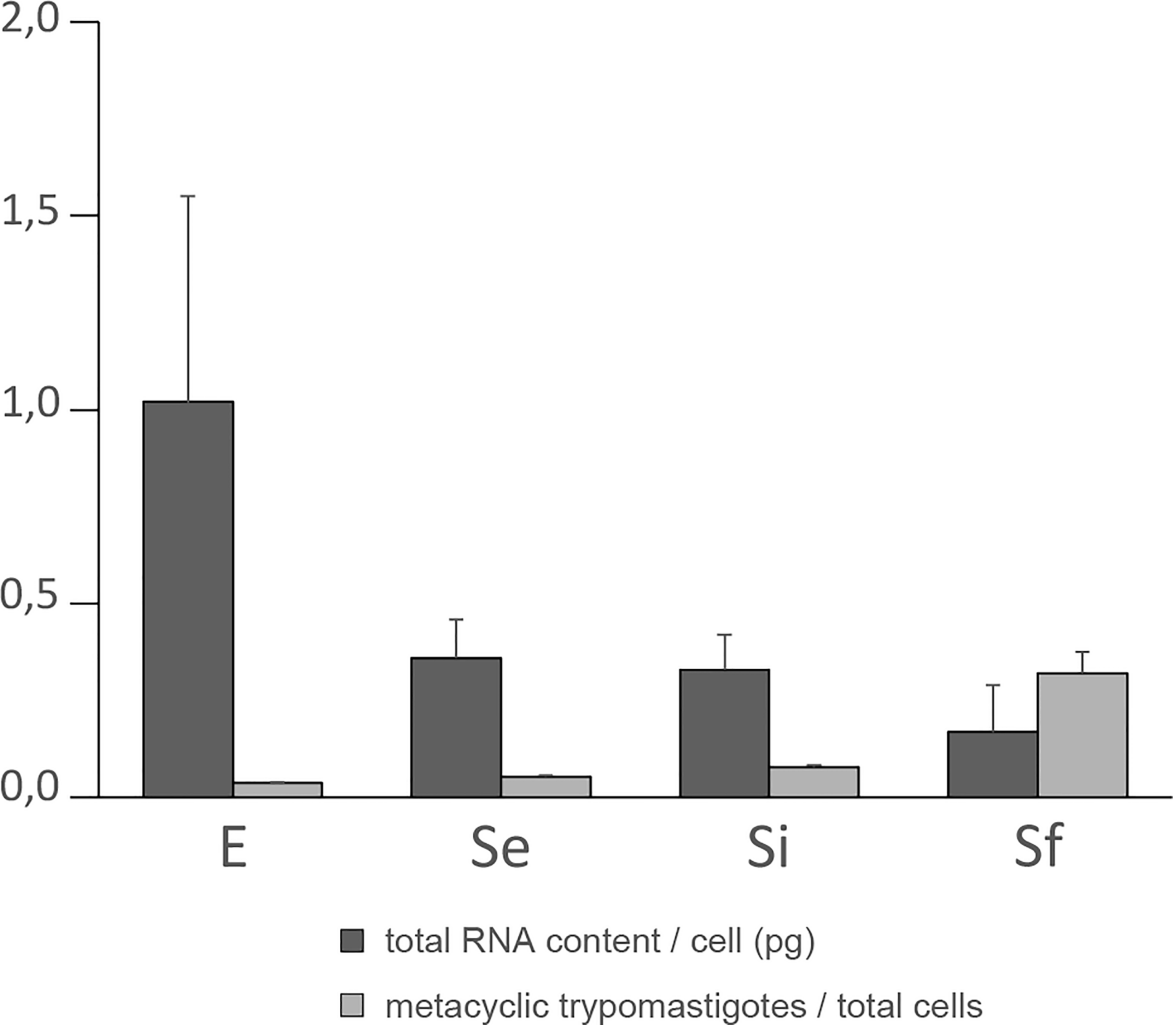
Total RNA content/cell and percentage of metacyclic trypomastigote during prolonged cultures of *T. cruzi* epimastigotes. Total RNA content/cell (pg) and % of metacyclic trypomastigotes of the parasite population of three independent biological replicates for each of the selected points of the epimastigote long-lasting growth curve: exponentially phase (E) and early, intermediate, and final stationary phase (Se, Si, and Sf respectively). Standard errors are indicated.

As a first step in transcriptomic analysis, the total RNA content per cell during the culture period was determined (**Figure 1**). The exponentially growing epimastigotes exhibit a total RNA content per cell (1.02 ± 0.53 pg) similar to previous reports in a different culture medium (0.6 ± 0.1 pg (Pastro et al., 2017)). As expected, the exponentially growing epimastigotes contain more total RNA per cell than the parasites in the stationary phase (0.36 ± 0.10 pg for Se). This value remains constant for the parasite population in the middle of the growth plateau (0.33 ± 0.09 pg for Si). Still, it diminishes even more at the beginning of the growth decline (0.17 ± 0.12 pg for Sf). Concordantly, transcription activity in exponentially growing epimastigotes has been estimated to be about six to ten times more active than in stationary cells (Nepomuceno-Mejía et al., 2010). In addition, under nutritional stress, an increase in the number of cytoplasmic granules (Santos et al., 2018), which are associated with mRNA degradation (Cassola et al., 2007; Holetz et al., 2007) has been described.

The increase in metacyclic trypomastigotes in the parasite populations may contribute to some extent to the observed changes in total RNA content per cell particularly for the final stationary phase (Sf). Meanwhile, a constant value expanding from the early to the intermediate stationary phase (Se to Si), clearly different from the exponentially growing parasite populations (E), was observed along the *T. cruzi* culture in nutrient-restricted conditions.

### Persistent expression changes are found in starving conditions of *in vitro* cultured *T. cruzi* epimastigotes

**Figure 2:**
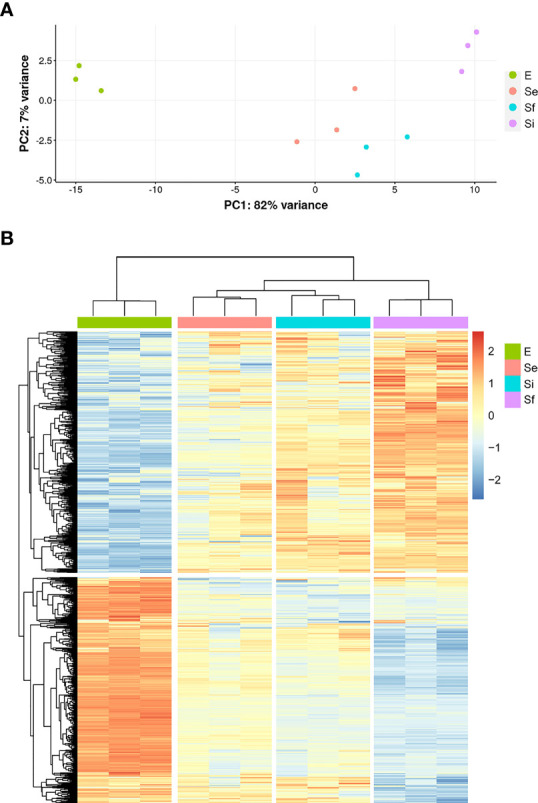
Transcriptomic data during prolonged cultures of *T. cruzi* epimastigotes. Analysis of transcriptomic data derived from polyA+RNA of three independent biological replicates of selected parasite population of the epimastigote long-lasting growth curve: exponentially phase (E) and early, intermediate, and final stationary phase (Se, Si, and Sf, respectively). **(A)** Principal Component Analysis of individual replicates. **(B)** Hierarchical clustering of differentially expressed genes.

RNA-seq analysis of three biological replicates for the parasite populations either in the exponential (E) or stationary phase at the three selected points (Se, Si, and Sf) was performed. Principal component analysis (PCA) of the transcriptomic data groups together the three independent biological replicates of each parasite population (**Figure 2A**). In addition, the first component, which explains more than 80% of the variation, clearly joins Se and Si and separates them from the exponentially growing epimastigotes (E) and the parasite population at critical nutrient restriction (Sf). This is further supported by the hierarchical cluster analysis of the differentially expressed genes (DEGs, 797 genes **Supplementary Table 1**), which joins Se and Si despite the gradual profile changes (**Figure 2B**). Indeed, neither up nor downregulated genes could be detected between Se and Si (log2FC, FDR < 0.05) (**Table 1**). Nonetheless, the number of differentially expressed transcripts gradually increases when comparing E to the successive stationary phase points analyzed (169, 311, and 691 DEGs for Se, Si, and Sf vs. E, respectively). Besides, a lower number of DEGs is observed when comparing Se or Si to Sf (88 and 77, respectively). A similar number of up and downregulated transcripts were observed within DEGs for the different comparisons.

**Table 1:** Number of significant up/downregulated genes at each analyzed time point.

	E	Se	Si	Sf
E		79	170	363
Se	90		0	53
Si	141	0		18
Sf	328	35	59	

The number of significantly up (above diagonal) or down (below diagonal) regulated genes at each of the stationary phase selected points: early (Se), intermediate (Si) and final stationary phase (Sf) is shown. The IDs of these differentially expressed genes are presented in **Supplementary Table S1**.

**Figure 3:**
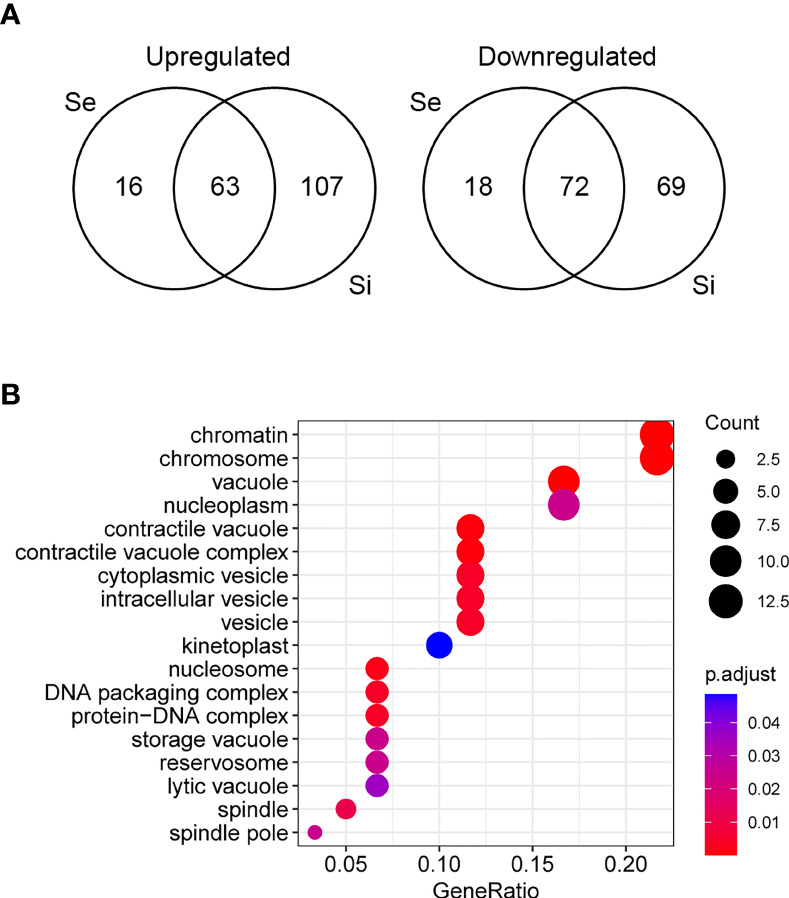
Analysis of differentially expressed genes of *T. cruzi* epimastigotes during prolonged cultures. The identified DEGs of early and intermediate stationary phase (Se and Si, respectively) in comparison to exponentially growing epimastigotes (E) were analyzed. **(A)** Venn diagrams. **(B)** Gene ontology analysis of downregulated Se⋂Si vs E DEGs.

Since the persistence of a homogeneous transcriptomic core (at least from Se to Si), clearly different from the exponentially growing parasite population (E), may reflect the molecular distinctiveness of *T. cruzi* parasites in prolonged culture conditions, we focused on the analysis of the shared DEGs of Se and Si vs. E (Se⋂Si DEGs). When E was used as reference, a high percentage (approx. 80%) of the Se DEGs were shared with Si (135 out of 169); instead, for Si, the percentage of DEGs shared with Se was lower (43,4%, 135 out of 311) (**Figure 3A**). Fewer DEGs were found when comparing the common DEGs between Se and Si vs. Sf (**Supplementary Table 1**). Interestingly, of the 4 possible intersections of the common up or downregulated genes of Se and Si vs E and Sf, only one (intersection of upregulated Se and Si vs E with downregulated Se and Si vs Sf) was not void. Two transcripts, coding for an NLI interacting factor-like phosphatase and a hypothetical protein with homology to cyclin10 (TcCLB.506525.120, TcCLB.503885.100) showed a significant increase in expression from E to Sf. The expression of both proteins is highly upregulated in metacyclic trypomastigotes (13 and 27 times, respectively) (Smircich et al., 2015).

The Se⋂Si DEGs include genes coding for hypothetical proteins whose proportion is similar to that of the whole genome (28%, 38 out of 135 being 19 genes upregulated, 10 conserved and 1 conserved pseudogene, and 19 genes downregulated, 17 conserved). Gene ontology analysis identified a single enriched category in the upregulated Se⋂Si DEGs: trans-sialidase activity 12/32, molecular function ontology, and three enriched categories in the downregulated Se⋂Si DEGs (**Figure 3B**): vacuole/vesicle 10/60, chromosome 13/60 and kinetoplast (6/60), cellular component ontology, which may be persistently acting under nutritional stress.

In accordance with omics approaches of epimastigotes in the early stationary phase (Santos et al., 2018), surface proteins are well represented among the upregulated Se⋂Si DEGs in our study (33%, 21 out of 63), accompanying the transition to metacyclic trypomastigotes. In addition to the 12 genes and 4 pseudogenes coding for proteins of the trans-sialidase superfamily of genes (TcTSs) identified in the gene ontology analysis (4 in Group II, 2 in Group IV, 3 in Group V, 1 in Group VII and 2 unclassified), 3 genes putatively coding for GP63, one for a mucin associated MASP, and a pseudogene for a type II mucin are also upregulated (**Supplementary Table 1**). Conversely, only two mucin genes are found among the downregulated Se⋂Si DEGs (TcCLB.506533.142, TcCLB.509147.50, belonging to the subfamily of mucins that are present at the surface of the epimastigote stage (TcSMUGL) [Urban et al., 2011)]. Surface proteins play different roles in the parasite’s life cycle progression, host-cell interplay, immune system evasion, and persistence of the parasite (Pech-Canul et al., 2017). To shed light on the development of the infectivity ability, we carried out a deeper analysis of the dynamics of these multi-gene families (TcTS, Mucins and DGFs) together with the analysis of other genes involved in the pathogenesis (to be published elsewhere). In view of the diversity of GO terms downregulated under our conditions, we further analyzed these genes to get insights into the molecular processes modulated.

The Se⋂Si DEGs code for enzymes that are themselves and/or their products involved in various cellular regulatory pathways. Cell organization is based on a complex metabolic network composed of numerous, sometimes redundant, but coordinated routes that grant homeostatic equilibrium. While the subcellular location and roles displayed by the Se⋂Si DEGs in response to nutrient restriction conditions must be investigated, we further analyzed the DEGs included in the major downregulated cellular components revealing potential biological roles.

### DEGs related to vacuole/vesicle functionality are found in starving conditions of *in vitro* cultured *T. cruzi* epimastigotes

The Se⋂Si DEGs downregulated in the enriched categories including vacuole and vesicle terms (see **Figure 3**) code for ten proteins that have been identified as associated with one or more different organelles (**Table 2**), including 4 (out of 71) associated with reservosome fractions (Sant’Anna et al., 2009) as well as 7 (out of 107) associated to the contractile vacuole fraction (Ulrich et al., 2011).

**Table 2:** Downregulated Se⋂Si DEGs in the enriched categories including vacuole and vesicle terms.

GeneID	Gene product	Product location
TcCLB.503893.30	hypothetical protein, conserved	CVC*
TcCLB.506893.100	UMP-CMP kinase, mitochondrial, putative	CVC*
TcCLB.506925.300	cyclophilin a, putative	Reservosomes*
TcCLB.508209.100	10 kDa heat shock protein, putative	CVC*
TcCLB.508719.30	hypothetical protein, conserved	CVC*
TcCLB.509445.10	tryparedoxin peroxidase, putative	Reservosomes*
TcCLB.509551.30	mitochondrial phosphate transporter, putative	CVC*
TcCLB.510101.140	pyruvate phosphate dikinase, putative	CVC*
TcCLB.510535.100	cysteine peptidase C (CPC), putative	Reservosomes*
TcCLB.510773.20	Vacuolar proton pyrophosphatase 1, putative	Reservosomes*, CVC*

The protein products have been identified as associated with one or more different organelles including reservosome fractions (Reservosomes*) (Sant’Anna et al., 2009) and contractile vacuole fraction (CVC*) (Ulrich et al., 2011).

Reservosomes are organelles dedicated to storing macromolecules ingested by endocytosis in *T. cruzi* epimastigotes (Soares and De Souza, 1988) that fuel the requirements of energy and amino acid dependence of metacyclogenesis and are not present as such in further developmental stages (Soares and de Souza, 1991; Sant’Anna et al., 2008; Vidal et al., 2017). During metacyclogenesis, increased cruzipain relocation to reservosomes and increased proteolytic activity induced by autophagy have been described (Soares et al., 1989; Alvarez et al., 2008; Vanrell et al., 2017; Losinno et al., 2021). It is worth noting that for the cysteine peptidase (**Table 2**), in addition to the proteolytic molecular function associated to the reservosomes, a role in the cell cycle and postranscriptional regulation of gene expression has been proposed for the *T. brucei* ortholog (Tb927.6.560) (Archer et al., 2011; Erben et al., 2014). In addition, both cyclophilin A and vacuolar proton pyrophosphatase 1 have been detected associated not only with reservosomes (**Table 2**) but also with chromatin (Leandro de Jesus et al., 2017). Finally, as its name reveals, this later protein has also been identified in an enriched contractile vacuolar fraction (Ulrich et al., 2011).

Contractile Vacuole Complexes (CVC) are organelles dedicated to regulating cell volume, present in several protists devoid of a cell wall, and are linked to acidocalcisomes, initially described in trypanosomatids but later found in many species (Docampo et al., 2005; Docampo et al., 2013; Jimenez et al., 2022). This compartmentalization constitutes a means to regulate osmotic pressure through cAMP signaling and microtubule-dependent fusion and the intracellular pH through alkalization/acidification, features that have also been implicated in parasite persistence, infection, and survival under starvation conditions (Galizzi et al., 2013; Mantilla et al., 2021). Nonetheless, the CVC is being recognized as a plastic organelle with pleiotropic functions in *T. cruzi*, including not only calcium homeostasis, cell volume and osmo regulation but also the trafficking functions in secretory and endocytic pathways involving the flagellar pocket, acidocalcisomes, mitochondria and the endoplasmic reticulum (Jimenez et al., 2022). Pyruvate phosphate dikinase is well known for its location and role in the ATP/ADP balance in glycosomes (Bringaud et al., 1998). The vacuolar proton pyrophosphatase 1 is one of the main pumps responsible for the acidocalcisomes acidification and may play a similar role in reservosomes. In *T. brucei*, acidification of acidocalcisomes is essential for initiating autophagy (Li and He, 2014), a critical process in metacyclogenesis (Vanrell et al., 2017). Globally these activities, which are preferentially located in these organelles, may modulate the rapid increase in short and long-chain polyPs levels detected during the lag phase of epimastigote growth (Ruiz et al., 2001). Related to cAMP signaling, the upregulated Se⋂Si DEGs of the *T. cruzi* parasites in starving conditions include four putative receptor-type adenylate cyclase (TcCLB.507465.10, TcCLB.507467.10, TcCLB.509267.3, TcCLB.509449.10). The increase in intracellular cAMP concomitantly with the upregulation of adenylyl cyclases expression (both mRNA and protein) together with their relocalization to the flagellum in response to nutritional stress has been reported (Hamedi et al., 2015). In addition, a serine-threonine protein kinase (TcCLB.510741.70) is also upregulated. Serine-threonine kinases are a group of enzymes that play crucial roles in cellular proliferation and differentiation (Orr et al., 2000) and are considered the major effectors of cAMP in eukaryotic cells (Lander et al., 2021). Conversely, the expression of a putative protein kinase (TcCLB.507837.20) is downregulated. Protein phosphorylation plays a significant role in cell signaling, gene expression, differentiation, and global control of DNA/RNA-mediated processes.

It is worth mentioning the presence of inositol-3-phosphate (IP3) synthase (TcCLB.503639.10) among the downregulated DEGs, despite not being detected in the enriched contractile vacuole fraction (Ulrich et al., 2011). Its product, IP3, plays a role in calcium homeostasis and osmoregulation of acidocalcisomes (Docampo and Huang, 2015). In addition, IP3 is involved either in surface protein synthesis and also regulation in *T. brucei*(Cestari, 2020; Cestari and Stuart, 2020) and carbon metabolism, which, together with the downregulated phosphomannose isomerase (TcCLB.503677.10), lies at the beginning of the major pathway, either modulating the contribution of the glucose 6-phosphate to all the inositol containing compounds, or the fructose 6-phosphate to all the mannose containing compounds. Similarly, the gene coding for the mevalonate-diphosphate decarboxylase (TcCLB.507993.330) is also downregulated. The mevalonate pathway is highly conserved and mediates the production of metabolites vital for cellular metabolism, growth, and differentiation, such as isoprenoids, which feed into biosynthetic pathways for sterols, dolichol, ubiquinone, heme, isopentenyl adenine, and prenylated proteins (Goldstein and Brown, 1990).

Acidocalcisomes also contain large amounts of amino acids, four times more concentrated than the whole cell, with 90% arginine and lysine likely as free amino acids and are not involved in the amino acid mechanism of volume regulation (Rohloff et al., 2003). Two genes putatively encoding enzymes involved in arginine metabolism: an amidinotransferase (TcCLB.505989.110) and an S-adenosylhomocysteine hydrolase, SAHH, (TcCLB.511589.200) are downregulated Se⋂Si DEGs. They may be acting to preserve arginine levels, which are similar in epimastigotes in exponential and stationary phases (Barisón et al., 2017). Another gene related to tyrosine metabolism: 2,4-dihydroxyhept-2-ene-1,7-dioic acid aldolase (TcCLB.503991.39), is also a downregulated Se⋂Si DEG. These DEGs plus the downregulated gene putatively coding for cytosolic malate dehydrogenase (TcCLB.506937.10) and the upregulation of several genes coding for amino acid permeases and transporters (5 putative permeases and one transporter (TcCLB.508923.10, TcCLB.511325.40, TcCLB.507811.100, TcCLB.511325.50, TcCLB.507101.10, TcCLB.511325.25) describe a global profile coinciding with the starvation-induced metabolic switch from glucose to amino acid consumption previously reported (Barisón et al., 2017). A wide range of biological functions has been proposed for cell or intracellular membrane transport proteins of kinetoplastids, establishing physiological properties such as membrane potential and responses to osmolarity, and in sensing and responding to changes in the environment (Landfear, 2019).

Related to autophagy, which has been involved in parasite survival during starvation and differentiation (Alvarez et al., 2008), the upregulation of a putative microtubule-associated protein 1A/1B light chain 3 (TcCLB.510533.180), a ubiquitin-like modifier involved in formation of vacuoles for autophagy was found. Nonetheless, we found the downregulation of a gene coding for a tubulin chaperone (tubulin binding cofactor A-like protein putative, TcCLB.509069.30) specific for the tight association of the alpha- and beta-tubulin subunits, as well as two genes for proteasome activator protein pa26, putative (TcCLB.503841.10, TcCLB.511001.240), with functional similarities to the eukaryotic PA28, a family of proteasome regulators whose members have controversial effects in promoting cellular growth and progression through the cell cycle. Their levels correlate with increased susceptibility to apoptosis, nuclear structure, and organization and effective DNA damage response, but specific functions in the context of adaptive cellular responses require further investigation (Cascio, 2021).

Interestingly, the gene coding for a protein 12 of the flagellum attachment zone (FAZ) (TcCLB.504153.260) is upregulated. FAZ is a morphogenetic structure with crucial regulatory roles in cell length and organelle positioning (Sunter and Gull, 2016) and its modulation has been involved in a variety of intermediate morphologies found under starvation conditions (Gonçalves et al., 2018).

In summary, long-term culture of *T. cruzi* epimastigotes without nutrient addition triggers the regulation of transcripts involved in specific vacuole/vesicle functionality, describing a complex metabolic network enabling the adaptation to the starving conditions while modulating the metacyclogenesis process.

### Starving conditions provoke expression changes of genes coding for proteins involved in the DNA/RNA functionality of *in vitro* cultured *T. cruzi* epimastigotes

The downregulated Se⋂Si DEGs in the chromatin/chromosome category (see **Figure 3B**) code for thirteen proteins that have been identified in a chromatin proteomic approach (Leandro de Jesus et al., 2017) (13 out of 60).

Four of these 13 genes are included in the category nucleosome/DNA packaging/protein-DNA complex. In addition, we find other 8 genes putatively coding for proteins related to this category, that were not identified using the above-mentioned chromatin approach. They include genes coding for a histone modification enzyme (Histone-lysine N-methyltransferase TcCLB.511417.70), a fragment of the retrotransposon hot spot protein (RHS) (TcCLB.511415.11) whose orthologue has been proposed to participate in control of chromatin structure and gene expression (Bernardo et al., 2020), two kinetoplast associated proteins (TcCLB.511039.10 and TcCLB.511529.80) which are involved in kDNA condensation and have been implied in cell proliferation and differentiation (de Souza et al., 2010), a chromosomal passenger complex 2 (TcCLB.506221.110) related to the regulate chromosome segregation and cytokinesis, a PIF1 helicase-like protein (TcCLB.506775.90) crucial for mitochondrial genome maintenance (Bochman et al., 2010; Liu et al., 2010), a DNA ligase (TcCLB.506287.209) and a mitochondrial DNA primase (TcCLB.503831.40).

The *T. brucei* ortholog of the product gene involved in RNA modification (**Table 3**) has been proposed to be a cytochrome oxidase subunit participating as an accessory factor in specific RNA editing (Sprehe et al., 2010). The other 8 genes (**Table 3**) code for proteins present in the chromatin proteomic approach and putatively code for proteins either of unknown function or that have been identified for DNA/RNA unrelated functions.

**Table 3:** Downregulated Se⋂Si DEGs in the chromatin/chromosome category.

GeneID	Gene product	Product function
TcCLB.506735.10	mitochondrial processing peptidase alpha subunit, putative	DNA/RNA unrelated functions
TcCLB.506925.300	cyclophilin a, putative	DNA/RNA unrelated functions*
TcCLB.507105.50	hypothetical protein	unknown
TcCLB.507817.18	histone H3, putative	nucleosome/DNA packaging/protein-DNA complex
TcCLB.508321.21	histone H2A, putative	nucleosome/DNA packaging/protein-DNA complex
TcCLB.508675.29	calpain-like cysteine peptidase, putative	DNA/RNA unrelated functions
TcCLB.508719.30	hypothetical protein, conserved	unknown **
TcCLB.509551.30	mitochondrial phosphate transporter, putative	DNA/RNA unrelated functions*
TcCLB.509965.290	p22 protein precursor, putative	RNA modification
TcCLB.510101.140	pyruvate phosphate dikinase, putative	DNA/RNA unrelated functions**
TcCLB.510525.80	histone H2A, putative	nucleosome/DNA packaging/protein-DNA complex
TcCLB.510525.90	histone H2A, putative	nucleosome/DNA packaging/protein-DNA complex
TcCLB.510773.20	Vacuolar proton pyrophosphatase 1, putative	DNA/RNA unrelated functions *,**

The protein products have been identified in chromatin proteomic approaches (Leandro de Jesus et al., 2017). Those also associated with other subcellular locations are indicated: *, reservosome fractions (Sant’Anna et al., 2009) and **, contractile vacuole fraction (Ulrich et al., 2011).

In addition to these 21 genes (13 identified by ontology analysis plus 8 through annotation characteristics), the downregulated Se⋂Si DEGs also include 6 genes related to DNA/RNA functionality: coding for ribosomal proteins (60S ribosomal protein L28, TcCLB.510101.40 and ribosomal protein S29 TcCLB.509201.15) and elongation factor (Elongation factor Tu, mitochondrial, TcCLB.506357.40); a mitochondrial RNA binding protein (TcCLB.510509.50), a poly(A) polymerase (TcCLB.510317.30) and an RNA binding protein (PSP1 C-terminal conserved region, TcCLB.506223.40).

Conversely only 6 of the upregulated Se⋂Si DEGs were found to be related to DNA/RNA functionality, including genes that putatively code for: an RNA binding protein (TcCLB.503683.30), whose ortholog in *T. brucei* has been proposed as an RNA expression modulator participating in the cell cycle (Erben et al., 2014; Lueong et al., 2016; Crozier et al., 2018), mismatch repair protein MSH4 (either a gene TcCLB.509967.20 and a pseudogene TcCLB.511911.30), that has been proposed to be involved in meiotic recombination based on its lack of an N-terminal mismatch interaction indicating the absence of function in the mismatch repair (Passos-Silva et al., 2010) as well as two small nuclear RNAs (snRNA U3, TcCLB.504427.244 and U6, TcCLB.503865.12) components of the spliceosome, which are transcribed by pol III, similarly as vault RNAs and other non-polyadenylated RNAs such as tRNAs, 5S rRNA, 7SL RNA (Kolev et al., 2019) with potential additional roles in regulation of various aspects of RNA biogenesis, from transcription to polyadenylation and RNA stability. Finally, we include in this subgroup the upregulated DEG (TcCLB.506203.10) coding for a nucleoside transporter which provides metabolic precursors for the synthesis of nucleic acids and energy metabolites as ATP and GTP, which are required for parasite viability in all life cycle stages because of the strict dependence on preformed purine salvage (Landfear et al., 2004; Boswell-Casteel and Hays, 2017).

Concordantly with the quiescent status, 20 out of the 135 Se⋂Si DEGs (fisher test p-value < 1e-13) were identified as peaking genes during different phases of the epimastigote cell cycle (Chávez et al., 2017) (**Supplementary Figure 1**). Only 2 are upregulated: one putatively coding for an amino acid permease (TcCLB.507811.100) and the other for a hypothetical protein (TcCLB.509203.14). The downregulated cell cycle peaking genes include two of the DEGs identified by ontology analysis (TcCLB.507105.50 and TcCLB.508321.21), the 8 DEGs identified by annotation terms, 4 DEGs coding for hypothetical proteins (TcCLB.506567.110, TcCLB.507809.39, TcCLB.506239.10 and TcCLB.507709.120) and 4 PAD (Protein Associated with Differentiation) DEGs putatively coding for proteins involved in different cellular functions: a cytosolic malate dehydrogenase (TcCLB.506937.10), a kinesin (TcCLB.506503.80), a nucleoporin NUP92 (TcCLB.504769.80) and an ESAG8-associated protein (TcCLB.511753.60). ESAG8-associated protein has been suggested to be involved in antigenic variation and development in *T. brucei*, and its ablation is related to the induction of the cell surface transporter PAD1 (Batram et al., 2014). PADs are surface protein markers that discriminate between the transmission stages in *T. brucei* (Dean et al., 2009). Interestingly, among the upregulated Se⋂Si DEGs, we identified PAD8 (TcCLB.509707.10). Most of the downregulated peaking genes (11 out of 18) are from the S phase, which corresponds to the DNA and organelle replication period.

In summary, nutrient depletion of *T. cruzi* epimastigotes triggers the downregulation of transcripts involved in cell proliferation and the control of chromatin structure. Nonetheless, complex regulatory networks that may affect not only the cell replication process but also different steps, such as mRNA maturation and translation, have been described. Interestingly, the up and downregulation of splicing ribonucleic factors may point to specific transcript processing control as previously described for mitochondrial RNA of nutrient-restricted *T. cruzi* epimastigotes (Shaw et al., 2016). The revealed up and downregulation of RNA binding proteins in response to nutrient depletion may account for the existence of active post-transcriptional regulons.

### A set of nuclear-encoded mitochondrial transcripts is downregulated in starving conditions of *in vitro* cultured *T. cruzi* epimastigotes

A high percentage of mitochondrial-related genes among the downregulated Se⋂Si DEGs was immediately noted. Thus, it was not surprising that kinetoplast appeared as an enriched term in the ontological analysis (see **Figure 3**).

**Figure 4:**
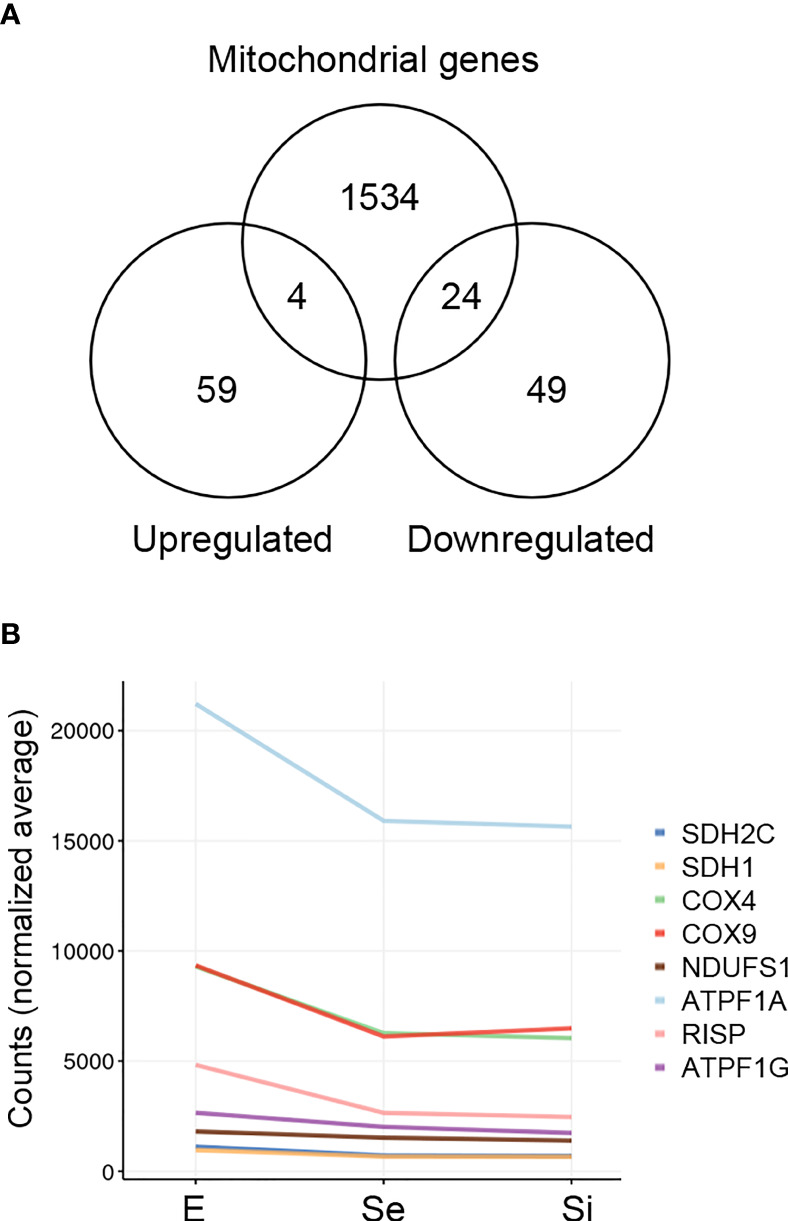
Mitochondrion-related DEGs of *T. cruzi* epimastigotes during prolonged cultures. The identified DEGs of early and intermediate stationary phase (Se and Si, respectively) in comparison to exponentially growing epimastigotes (E) were analyzed. **(A)** Venn diagrams comparing the Se⋂Si vs E DEGs with the genes with mitochondria term in TriTrypDB. **(B)** Spaghetti plot of nuclear genes encoding predicted mitochondrial proteins whose expression changes in starving conditions were previously reported (Shaw et al., 2016).

In order to obtain a thorough inventory of the mitochondrial-related genes in the Se⋂Si DEGs, we extracted the genes with the term “mitochondria” in the TriTrypDB, mainly expanded by the database of nuclear genes encoding predicted mitochondrial proteins, MiNT, we have previously reported (Becco et al., 2019). We found 28 out of 135 Se⋂Si DEGs upon nutrient restriction code for mitochondrial products (**Figure 4A**). Most of them (24 out of 28) are downregulated, whereas only four are upregulated. Among the Se⋂Si DEGs, in addition to these 28 DEGs, we noted the presence of a gene coding for a putative NADH-cytochrome b5 reductase (TcCLB.511047.40) which is also downregulated. Interestingly, overexpression of a cytochrome b5 reductase-like protein causes the loss of kinetoplast DNA in *T. brucei* (Motyka et al., 2006).

The downregulation of several nucleus-encoded components of the electron transport chain has been previously reported (Shaw et al., 2016) and is here confirmed (**Figure 4B**).

The four upregulated Se⋂Si DEGs coding for mitochondrial products include an ascorbate peroxidase (TcCLB.503745.30) that participates in oxidative damage prevention and is associated with the microsomal fraction in *T. cruzi* and is therapeutically exploitable because of its absence in the human host (Wilkinson et al., 2002), a putative DNAJ (TcCLB.508989.60) and two receptor-type adenylate cyclase (TcCLB.509267.3, TcCLB.509449.10).

Meanwhile, among the downregulated Se⋂Si DEGs coding for mitochondrial products, four were identified in the ontology analysis with the cellular component term: vesicle/vacuolar, including the genes coding for the hypothetical protein (TcCLB.503893.30), the mitochondrial UMP-CMP kinase (TcCLB.506893.100), the co-chaperone Hsp 10 (GroES) (TcCLB.508209.100) and the mitochondrial phosphate transporter (TcCLB.509551.30). Summing to this latter function, we identified another DEG coding for a putative mitochondrial cation transporter (TcCLB.509197.39). Besides, five additional genes coding for conserved hypothetical proteins (TcCLB.458015.4, TcCLB.504051.49, TcCLB.506289.30, TcCLB.507083.40 and TcCLB.507105.50) appeared among the downregulated Se⋂Si DEGs coding for mitochondrial products, supporting their involvement in the mitochondrial response to nutrient restriction. With the chaperone function, in addition to the co-chaperone Hsp 10 (GroES), we also found the downregulation of a gene putatively coding for a rotamase (TcCLB.510259.50) (Perrone et al., 2018) and a mitochondrial GrpE (TcCLB.507929.20) which is classically known to help fold proteins newly imported into mitochondria in conjunction with Hsp10 and Hsp60 (Fernandes et al., 2005) and may act in concurrence with DNAJ (Tibbetts et al., 1998). Although the expansion of the DNAJ family in kinetoplastids may support a diversification of roles in cellular homeostasis (Folgueira and Requena, 2007; Urményi et al., 2014), the presence of various components of the DNAJ/GRPE may support their suggested role in mitochondrial DNA maintenance and replication (Týč et al., 2015). As mentioned above, several Se⋂Si downregulated DEGs code for mitochondrial proteins involved in these processes and/or in DNA/RNA functionality (TcCLB.503831.40, TcCLB.506287.209, TcCLB.506357.40, TcCLB.506735.10, TcCLB.506775.90, TcCLB.509965.290, TcCLB.510509.50, TcCLB.511039.10 and TcCLB.511529.80) and in part support the reported effects of nutrient restriction on the mitochondrion of *T. cruzi* epimastigotes (Shaw et al., 2016; Pedra-Rezende et al., 2021).

**Figure 5:**
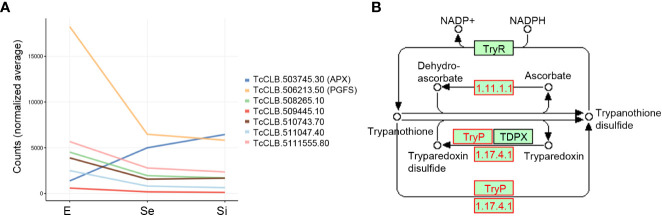
Trypanothione metabolism related DEGs of *T. cruzi* epimastigotes during prolonged cultures. Selected DEGs related to trypanothione metabolism of early and intermediate stationary phase (Se and Si respectively) in comparison to exponentially growing epimastigotes (E) were analyzed. **(A)** Gene expression of selected DEGs is presented as a spaghetti plot from Se and Si. TcCLB.503745.30: ascorbate peroxidase, TcCLB.506213.50: prostaglandin F synthase reductase, TcCLB.508265.10: glutathione-S-transferase/glutaredoxin, TcCLB.509445.10: tryparedoxin peroxidase, TcCLB.510743.70: lactoylglutathione lyase-like protein glioxilase, TcCLB.511047.40: NADH-cytochrome b5 reductase, TcCLB.511555.80: ribonucleoside-diphosphate reductase small chain. **(B)** Metabolic chart of the trypanothione reduction pathways indicating the enzymes involved. In red enzymes Ribonucleoside-diphosphate reductase large chain (EC: 1.17.41): TcCLB.506933.20, TcCLB.511555.80, TcCLB.506933.20, TcCLB.511555.80; Tryparedoxin peroxidase (EC:1.11.1.-): TcCLB.509445.10; TcCLB.509445.10; Ascorbate-dependent peroxidase (EC:1.11.1.11): TcCLB.503745.30.

Finally, a group of Se⋂Si putatively code for mitochondrial products involved in redox metabolism, including the upregulated ascorbate peroxidase and the downregulated NADH-cytochrome b5 reductase (TcCLB.511047.40) already mentioned, a prostaglandin F synthase reductase (pseudogene, TcCLB.506213.50) and a glutathione-S-transferase/glutaredoxin (TcCLB.508265.10) (**Figure 5A**). Outside the mitochondrial compartment, the gene putatively coding for the cytosolic malate dehydrogenase mentioned above, and at least three more genes coding for proteins involved in alternative pathways where the reduced form of trypanothione is consumed, are downregulated: a lactoylglutathione lyase-like protein glyoxalase (TcCLB.510743.70) (Sousa Silva et al., 2012), a ribonucleoside-diphosphate reductase small chain (TcCLB.511555.80), and the tryparedoxin peroxidase already presented with the vacuolar term. Globally these findings may indicate an increased role of the ascorbate peroxidase in maintaining the oxidized status of trypanothione while the pathways involved in the detoxification of oxoaldehydes, metals, and drugs in the synthesis of deoxyribonucleotides and decomposition of peroxides are decreased (**Figure 5B**). Interestingly, we did not find an increase in transcripts for ascorbate peroxidase in metacyclic trypomastigotes (Smircich et al., 2015), suggesting that the upregulation of this enzyme may be a characteristic of the transitional parasites in the early and intermediate stationary phase.

### Distinctive gene expression of *T. cruzi* epimastigotes in starving conditions

To identify genes that characterize the transitional forms we searched upregulated Se⋂Si DEGs whose transcriptome values in metacyclic trypomastigote were lower than that of the exponentially growing epimastigotes from our previous data (Smircich et al., 2015); thus, the upregulation cannot be attributable to the metacyclogenesis process. This is the case for the expression profile exhibited by the ascorbate peroxidase (see above). In addition, we looked for the complementary profile, v.g. downregulated Se⋂Si DEGs whose transcriptome values in metacyclic trypomastigote were higher than that of the exponentially growing epimastigotes from our previous data (Smircich et al., 2015); thus, the downregulation cannot be attributable to the metacyclogenesis process.

**Figure 6:**
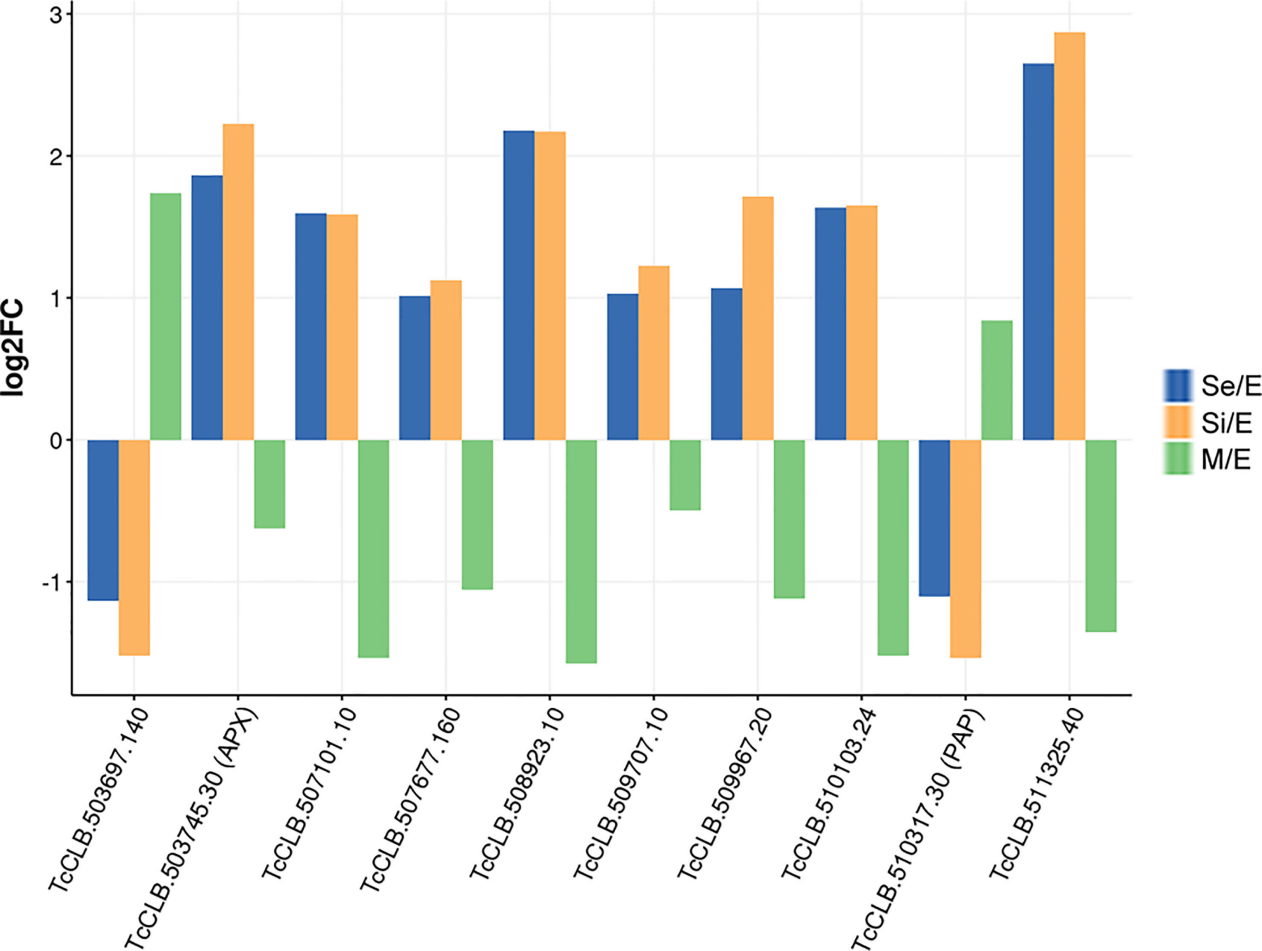
DEGs of *T. cruzi* epimastigotes during prolonged cultures with a distinctive expression profile either from exponentially growing epimastigotes or metacyclic trypomastigotes. The expression profile of DEGs of early and intermediate stationary phase (Se and Si, respectively) in comparison to exponentially growing epimastigotes and of DEGs of metacyclic trypomastigotes (M) in comparison to exponentially growing epimastigotes from our previous data (Smircich et al., 2015) were analyzed to identify distinctive expression profile of starving parasites either from epimastigotes or metacyclic trypomastigotes.

We found that at least 10 genes can be distinctively attributed to transitional parasite forms enriched by nutrient restriction (**Figure 6** and **Supplementary Figure 2**). In addition to the gene coding for the ascorbate peroxidase, two genes encoding conserved hypothetical proteins are upregulated exclusively during the starvation period (TcCLB.507677.160 and TcCLB.510103.24). Both orthologs have been described as nodulin-like and/or as members of the major facilitator superfamily of transporters (MFS). Nodulins were first described in legumes and are specifically expressed during the development of symbiotic root nodules (Legocki and Verma, 1980). Nonetheless, their importance for the transport of nutrients, solutes, amino acids or hormones, and plant development, even in non-nodulating plants, has been widely recognized (Denancé et al., 2014). Meanwhile, MFS is the largest known superfamily of secondary active transporters, responsible for carrying a broad spectrum of substrates (for a review, see (Drew et al., 2021)). While TcCLB.507677.160 orthologs in *T. brucei* are related to pyruvate transport, an essential pathway in parasite differentiation (Sanchez, 2013), TcCLB.510103.24 is related to proteins associated with PADs which, as previously mentioned, are markers of parasite differentiation in *T. brucei* (Dean et al., 2009). Besides, genes putatively coding for three amino acid permeases (TcCLB.507101.10, TcCLB.508923.10, and TcCLB.511325.40), a protein associated with differentiation PAD8 (TcCLB.509707.10) and a mismatch repair protein MSH4 (TcCLB.509967.20) showed distinctive upregulated expression during the starvation period. On the other hand, two genes encoding a conserved hypothetical protein TcCLB.503697.140, and a poly(A) polymerase, TcCLB.510317.30, are downregulated during the starvation period while upregulated during metacyclogenesis.

Only four of these 10 genes (TcCLB.509967.20, TcCLB.510317.30, TcCLB.507677.160, TcCLB.509707.10) have homologs in the *T. brucei* genome. Members of the families of homologs of two of them (pyruvate transporter TcCLB.507677.160 and PAD gene families TcCLB.509707.10) have been proposed as markers of differentiation clusters obtained by single-cell RNA-seq (Briggs et al., 2021; Hutchinson et al., 2021; Howick et al., 2022). The fact that in Leishmania all 10 genes have homologs may suggest more similar differentiation processes in these parasites with intracellular stages. Even though the number of the here-disclosed DEGs is low, they contrast the presumptive progressive transcriptomic changes from epimastigotes to metacyclic trypomastigotes. The set of genes revealed here may be considered as a molecular signature of the transitional parasite form that *T. cruzi* epimastigotes adopt under starvation conditions.

## Conclusions

Different *in vitro* approaches, resembling the progressively nutrient-restricted environment *T. cruzi* deals with inside the digestive tract of the vector, have contributed to understanding several aspects of the insect-dwelling differentiation process.

Understanding the molecular mechanisms underlying the differentiation process in this unicellular organism that is able to rapidly adapt to several host-changing environments may contribute to shedding light on the complex biological processes that individual cells may display in response to different host stimuli.

Aiming to reveal genes distinctly expressed by *T. cruzi* in the starving periods that the insect host undergoes, we analyzed the transcriptomic changes of epimastigotes maintained in culture for extended periods without media refreshment leading to nutrient restriction. Genes related to surface proteins, the management of protein quality or content, the reported switch from glucose to amino acid consumption and the redox challenge were found to be differentially expressed. Control of the adaptation to nutrients restriction relies on regulated metabolic and molecular processes, including DNA, RNA, and histone modification pathways, which in turn depend on the enzymes that catalyze these modifications as well as in the transport and production of metabolites generated in specific compartments of the cell. Contractile vacuole, and reservosomes appeared as cellular components in the enriched vacuole/vesicle ontology terms, highlighting the relevant roles of these organelles in cell volume, osmoregulation and metabolic homeostasis in starving conditions. Whether these changes are part of a pre-established differentiation program triggered in our conditions or a response to specific stimuli in our culture remains to be studied. Although ontology analysis is biased by the available data, the detailed revision of DEGs further supports the obtained results. Concordantly with the quiescent status derived from nutrient restriction, cell cycle peaking genes were enriched among the Se⋂Si DEGs). In addition, we observed a remarkable number of differentially expressed genes related to the unique parasite mitochondrion, that may be coordinately acting with intramitochondrial regulation of expression to alleviate the nutritional restriction stress.

Remarkably, some of the differentially expressed genes remain stable in the stationary phase versus exponentially growing epimastigotes and do not follow the expected metacyclogenesis profile. This finding could be considered consistent with the proposed existence of an intermediate stage between epimastigotes and metacyclic trypomastigotes. Particularly, the distinctive expression profiles of genes related to transport and differentiation were revealed. Interestingly, homologous genes in *T. brucei* have been identified as important markers of differentiation in recent scRNA-seq experiments. Similar work on *T. cruzi* facilitated by single-cell approaches may enrich the data presented here.

Comparative transcriptomic approaches performed in parasites obtained from insect hosts during the starvation period may provide further insights. Besides, novel technologies that enable 3D cell cultures to simulate the environment of specific organs, just emerging in the field of trypanosomatids (Sutrave and Richter, 2021), may facilitate the host interaction analysis. Nevertheless, the gene expression changes here presented, triggered and persistently maintained in epimastigotes culture by starvation and associated physicochemical changes may support the existence of a transitional parasite form in *T. cruzi* life cycle between epimastigotes and metacyclic trypomastigotes in the insect host.

## Data availability statement

The datasets presented in this study can be found in online repositories. The names of the repository/repositories and accession number(s) can be found below: NCBI, BioProject ID PRJNA915394.

## Author contributions

PS and BG conceived aims and strategies. PS, LP-D, MD, and BG designed the experiments. LP-D and FH performed parasite culture and quantification, RNA extraction, and quantification. PS performed the bioinformatic analysis. PS and BG analyzed the data. PS and BG wrote the first draft of the manuscript. All authors contributed to manuscript revision, read, and approved the submitted version.
