# Supplementary material for: Transcriptomic analysis of the adaptation to prolonged starvation of the insect-dwelling Trypanosoma cruzi epimastigotes
Source: Front Cell Infect Microbiol. 2023 Apr 6;13:1138456. doi: 10.3389/fcimb.2023.1138456 (PMC10117895; doi:10.3389/fcimb.2023.1138456)
Supplement: Supplementary file 1 [file Table_1.docx]

Supplementary Material

Transcriptomic analysis of the adaptation to prolonged starvation of the insect-dwelling Trypanosoma cruzi epimastigotes

Pablo Smircich^1,2*^, Leticia Pérez-Díaz^1^, María Ana Duhagon^1,3^, Beatriz Garat^1*^

*** Correspondence:** Corresponding Author: [bgarat@fcien.edu.uy](mailto:bgarat@fcien.edu.uy), [psmircich@fcien.edu.uy](mailto:psmircich@fcien.edu.uy)

# Supplementary Figures and Tables

## Supplementary Figures

**

**

**Supplementary Figure 1.** Venn diagrams of upregulated and downregulated differentially expressed genes of *T. cruzi* epimastigotes in starving conditions (early and intermediate stationary phase, Se and Si respectively) in comparison to exponentially growing epimastigotes (E), Se⋂Si vs E DEGs, and peaking genes along the epimastigote cell cycle (Chávez et al., 2017).


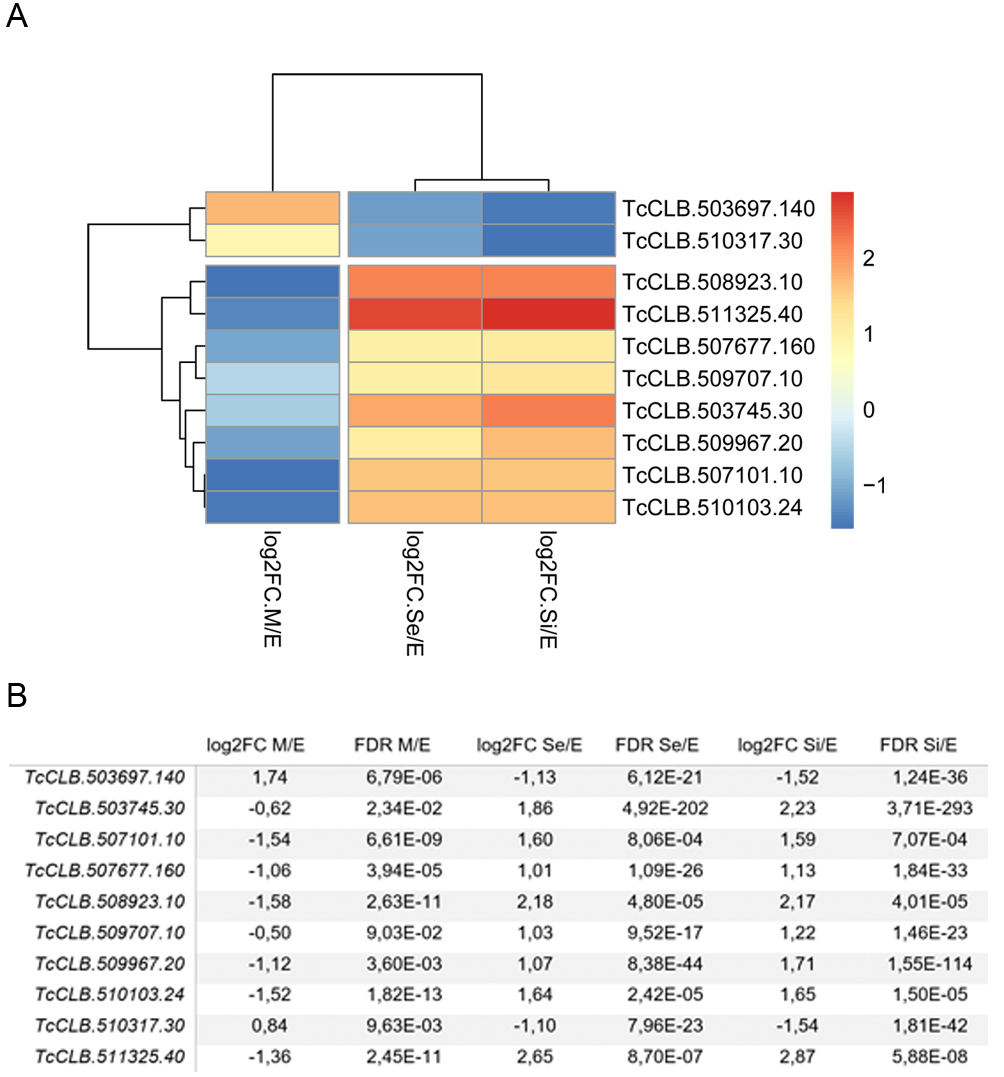


**Supplementary Figure 2.** **Analysis of differentially expressed genes of T. cruzi epimastigotes in starving conditions with a distinctive expression profile from either exponentially growing epimastigotes and metacyclic trypomastigotes.** The expression profile of DEGs of early and intermediate stationary phase (Se and Si respectively) in comparison to exponentially growing epimastigotes and of DEGs of metacyclic trypomastigotes (M) in comparison to exponentially growing epimastigotes from our previous data (Smircich et al., 2015) were analyzed to identify distinctive expression profile of starving parasites either from epimastigotes or metacyclic trypomastigotes **(A)** Heatmap **(B)** Fold change and FDR for each gene in A.
